# Knowledge, Attitudes, and Behaviors Related to Dementia Prevention and Caregiving Among Korean Americans (the KIMCHI Project): Pre- and Posttest Evaluation Study

**DOI:** 10.2196/72147

**Published:** 2025-08-15

**Authors:** Van Ta Park, Bora Nam, Daren Huang, Stacy W Yun, Nicole Phan, Eun Jeong Lee, Hye-Won Shin

**Affiliations:** 1 Department of Community Health Systems School of Nursing University of California, San Francisco San Francisco, CA United States; 2 Psychology Department University of Colorado Colorado Springs Colorado Springs, CO United States; 3 Asian American Resource and Information Network, Inc Wood Ridge, NJ United States; 4 Department of Human Services NYC College of Technology City University of New York New York, NY United States; 5 Somang Society Cypress, CA United States; 6 University of California, Irvine Institute for Memory Impairments and Neurological Disorders Irvine, CA United States

**Keywords:** Alzheimer disease and related dementias, Korean Americans, knowledge, attitudes, behaviors, caregiving, community-based dissemination

## Abstract

**Background:**

Many Korean American older adults have limited English proficiency, have low socioeconomic status, and are immigrants. The availability and accessibility of linguistic and culturally appropriate dementia-related health care and caregiving resources for this population are limited. This is concerning given that Korean American older adults are a rapidly growing population, and Alzheimer disease and related dementias (ADRD) represent a significant public health issue.

**Objective:**

We aimed to assess changes in pre- and posttest knowledge, attitudes, and behaviors regarding ADRD prevention and caregiving among participants in the Koreans Invested in Making Caregivers’ Health Important (KIMCHI) before and after their participation in KIMCHI workshop presentations.

**Methods:**

A community engagement dissemination project, KIMCHI, was developed by academic and community partners to culturally and linguistically tailor selected evidence-based research from the Patient-Centered Outcomes Research Institute for Korean American older adults, caregivers, and other stakeholders. Dissemination activities were conducted in-person (as workshops) and on digital platforms. Through partnerships with 1 academic institution and 2 community organizations that serve Korean Americans, 211 participants participated in the KIMCHI in-person workshop presentations, and 134 participants participated asynchronously online (via fact sheets on the project website, YouTube videos, and other social media, such as Facebook and X [formerly known as Twitter]). Pre- and postparticipation tests on knowledge, attitudes, and behaviors for ADRD and caregiving were conducted with workshop participants. We administered satisfaction surveys to all participants (workshop and online) and conducted two-tailed paired-sample 2-tailed *t* tests to assess mean changes in the pre- and postparticipation tests.

**Results:**

Among the workshop participants (N=211), most (114/204, 55.9%) were older adults (mean age 69, SD 12.1 y; range 24-90 y), female (n=148, 70.1%), and foreign-born (n=203, 96.2%). Many reported having limited English proficiency (167/211, 79.1%). Significant changes were observed in posttest ADRD knowledge (mean 11.51, SD 2.64), attitudes (mean 7.13, SD 2.83), and behaviors (mean 8.88, SD 2.72) compared to pretest scores (knowledge: mean 10.34, SD 2.67; *t*_210_=1.17; *P*<.001; attitudes: mean 6.33, SD 2.44; *t*_210_=0.8; *P*<.001; and behaviors: mean 8.11, SD 2.68; *t*_210_=0.76; *P*<.001). Workshop participants reported high satisfaction (196/209, 93.8%) with KIMCHI, with the workshop presentations being perceived as culturally relevant and applicable (196/209, 93.8%). Most workshop participants expressed interest in learning more about ADRD-related health topics (186/209, 89%). Similarly, the online participants (N=134) expressed high satisfaction (115/134, 85.8%) and agreed that the topics and content were culturally relevant and applicable (116/133, 87.2%) and learned new information (110/134, 82.1%).

**Conclusions:**

The findings indicate that KIMCHI may have a positive impact on improving ADRD knowledge, attitudes, and behaviors among Korean Americans. Academic-community collaborations should continue to culturally tailor the programs and studies to help ensure greater representation of Korean Americans in research and community engagement projects.

## Introduction

### Background

According to the US Census Bureau and Pew Research Center data, Korean Americans, who encompass 8% of all Asian American subgroups, have rapidly expanded to approximately 2 million in the United States as of 2023 and are the fifth largest Asian origin in the United States [1,2]. Following the Immigration and Nationality Act of 1965, which removed national immigration quotas, the Korean immigrant population steadily rose, contributing to the development of Korean communities across the United States [3].

Despite their growing presence, Korean Americans face a range of challenges on dementia-related knowledge gaps. The 2015 Asian American Quality of Life survey, which included 471 Korean Americans, found that lack of literacy led to concerns, worries, and unpreparedness if one has Alzheimer disease (AD) or is a caregiver for individuals with AD [4]. In addition, researchers who conducted a cross-sectional survey of AD knowledge among Korean Americans (N=268) in the Greater Washington metropolitan area found the main topic participants reported to be the least knowledgeable about was caregiving [5]. In a scoping review that examined the caregiving experiences of Korean American caregivers of persons living with dementia [6], the authors described how caregivers experience challenges in finding culturally relevant AD and related dementias (ADRD) resources, which limits their ability to learn new information about dementia caregiving.

Dementia remains a major health concern among older Korean American adults and their families. Studies indicate that Korean American caregivers experience higher levels of caregiving distress and fewer coping mechanisms compared to the general population, partly due to a common belief that dementia is a natural part of aging, which discourages seeking support [7,8]. Notably, Korean American caregivers have difficulty in confronting their family members about memory loss due to stigma associated with memory loss and the belief that it is better to keep the issue in the family [7]. Koreans living in the United States tend to engage in family caregiving rather than relying on formal support services [9]. In addition, only 7% of older Korean Americans with probable dementia seek medical advice [10]. While informal social support helps reduce caregiving stress, these findings underscore the need for culturally appropriate dementia education and support within this community [11]. While caregiver stress is a concern, this project does not directly measure or address it. However, to bridge the dementia-related knowledge gaps, we launched the Koreans Invested in Making Caregivers’ Health Important (KIMCHI) project, a national bilingual project designed to address the misconceptions about ADRD and empower Korean Americans with information on healthy cognitive aging and caregiving resources. To the best of our knowledge, there is 1 previous educational study [12] of Korean Americans with dementia and their caregivers; however, the study did not implement an immediate pre- and posttest approach nor include a digital platform for engagement. By incorporating core Korean cultural values that emphasize family-based support, KIMCHI aims to provide tools for informed health care decision-making while respecting these cultural traditions. We hypothesized that participation in the KIMCHI project would lead to improved ADRD-related knowledge, attitudes, and behaviors (KAB) among Korean American participants, and an increased willingness to learn more about ADRD and be involved in future projects supported by the Patient-Centered Outcomes Research Institute (PCORI).

### Objectives

KIMCHI was supported by a PCORI community dissemination award to share recent patient-centered research findings with the Korean American community (eg, caregivers, older adults, and general public) in culturally and linguistically appropriate ways, as well as to enhance participants’ KAB regarding ADRD, healthy brain aging, and caregiving before and after attending the KIMCHI workshop presentations.

## Methods

### Design and Methods

**Figure 1:**
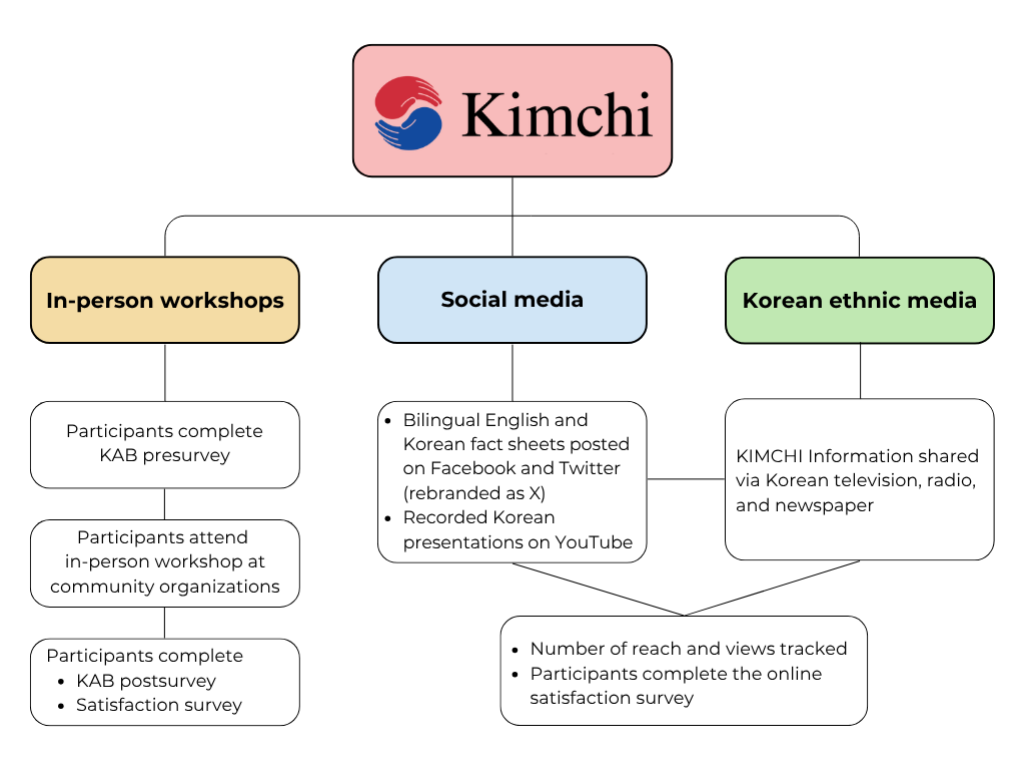
Flowchart of the 3 main dissemination strategies of the Koreans Invested in Making Caregivers' Health Important (KIMCHI) project. KAB: knowledge, attitudes, and behaviors.

The KIMCHI project culturally and linguistically tailored PCORI evidence findings and then disseminated this to Korean American stakeholders, such as patients with dementia, family members, caregivers, health care providers, and community-based organizations. Our dissemination strategy involved 3 main approaches (Figure 1; Textbox 1).

**Dissemination strategies.**

**In-person workshops**

- We presented the content via in-person workshops that were conducted in Korean. We administered pretest, posttest, and satisfaction surveys to the participants.

**Social media**

- We posted the bilingual English and Korean fact sheets on Facebook and Twitter (rebranded X), as well as the recorded Korean presentations on YouTube. We examined engagement via the number of views on YouTube, Facebook, and Twitter (rebranded as X).

**Korean ethnic media**

- We shared information about the Koreans Invested in Making Caregivers' Health Important (KIMCHI) project (eg, what it is, website, bilingual fact sheets, as appropriate) via major Korean media, including television, radio, and newspapers. The ethnic media provided us with the estimated reach for each type of medium. For both social and ethnic media, we encouraged the viewers and listeners to complete the satisfaction survey after reviewing the bilingual fact sheet.

### Participants and Recruitment

#### Overview

Participants were eligible to participate in KIMCHI activities if they identified as Korean American, were aged ≥18 years, and could read or speak Korean or English. Although ADRD predominately affects older individuals and caregivers, an adult of any age may be a caregiver to older adults (eg, grandparent), particularly among Asian American (including Korean American) families who live in multigenerational households (24% vs 13% White individuals in 2021) [13] and who are family-centric. For in-person project activities, participants were either asked to confirm their eligibility by phone or email before the scheduled activities. Recruitment efforts primarily leveraged community-based organizations, such as the Somang Society and the Asian American Resource and Information Network (AARIN), that serve Korean Americans in the United States. In addition to these organizations, recruitment strategies included flyers; Korean ethnic media outlets (major Korean television channels, Seoul Broadcasting System-USA, The Korean Channel news, and Radio Korea); social media; and word-of-mouth referrals. KIMCHI was also shared with the Korean participants in the Collaborative Approach for Asian Americans, Native Hawaiians, and Pacific Islanders (AANHPI) Research and Education registry, a registry aimed toward improving the representation of AANHPI populations in aging, ADRD, and caregiving research [14,15].

Initially designed to include 4 web-based presentations, the project expanded based on community feedback and technological barriers faced by older adults. The community’s main counterargument to our initial web-based presentation was that potential Korean American participants would have trouble joining Zoom on their phone or computer, and participants stated that “they do not want to have to bother their children or grandchildren to assist them due to their lack of technological knowledge,” which will deter them from enrolling into KIMCHI. Therefore, the KIMCHI team mitigated these barriers and hosted in-person workshops to accommodate the Korean American community.

#### Selected PCORI Evidence

The KIMCHI project was primarily supported by a PCORI community engagement dissemination award, which supports projects that help organizations and communities share pertinent PCORI-funded research findings to patients, families, caregivers, and the broader health care community in ways that will command their attention and interest and encourage use of this information in their health care decisions [16].

Findings from 2 PCORI-funded studies [17,18] informed our community education efforts to reduce stigma and facilitate discussions on ADRD caregiving. The rationale for selecting these studies was that they were the most relevant educational material on ADRD caregiving for Korean Americans among the list of potential PCORI-funded studies to disseminate. In the studies by Smith [17] and Jennings et al [18], the authors recommended that patients with dementia identify key personal goals (physical functioning, meaningful activities, and not being a burden to family) and prioritize these goals to improve their well-being. To do that, there has to be community education on dementia that informs caregivers how to set goals, including (1) the safety of patients with dementia, (2) goals with health care providers, and (3) managing caregivers’ stress [18]. The patients and caregivers need to readdress the goals of both sides if the patient with dementia progresses, articulating the need for caregivers to adapt and change their goals to accommodate the patient [18]. The KIMCHI project helps to provide caregivers with the necessary knowledge and skills to better care for their loved ones. In addition, both studies emphasized the need to provide community members with knowledge on healthy cognitive aging and what can be done to prevent dementia by offering examples of physical exercise, brain fitness, and social engagements [17,18]. Thus, as an example, the KIMCHI project informs the participants of (1) the knowledge component, which is the positive benefits of physical exercise, brain fitness, and social engagement for ADRD prevention; (2) the attitudes component, where examples of physical exercise, brain fitness, and social engagement activities will be discussed, which will affect their attitude or intention to practice these activities; and (3) behaviors component, where participants will indicate a plan or intention to change their behavior.

Additional studies also emphasized the benefits of behavioral interventions for caregivers and patients with dementia. Research on cognitive aging, dementia prevention, and goal setting for patients and caregivers highlights evidence-based strategies, including physical exercise and cognitive training for social and emotional quality of life, potentially reducing the dementia risk [17,18]. In addition, findings on dementia prevention interventions, such as wellness activities and yoga, improve the mood and lower the burden of caregivers and their partners with amnestic mild cognitive impairment [19]. On the basis of these findings, our dissemination content emphasizes brain health and dementia prevention strategies, outlining risk factors and protective factors while reinforcing a holistic approach to cognitive aging, conveying the message that what is good for the heart is good for the brain. In addition, it highlights the need for community support and culturally tailored education for Korean Americans. We also focus on 5 key domains in dementia caregiving—medical care, physical and emotional well-being, access to services, and caregiver support—stressing the importance of safety, stress management, and meaningful activities.

#### Cultural and Linguistic Tailoring

A culturally sensitive dissemination strategy was used, including bilingual materials and messaging tailored to the Korean American community. Initially, the KIMCHI team drafted dissemination materials, including presentation slides and fact sheets, in English. The materials were reviewed by the KIMCHI-wide team, including scientific advisers, who provided feedback on the accuracy, clarity, and interpretation of the PCORI research findings; their input informed several key improvements, such as replacing technical terms such as “psychotherapy” with more accessible language such as “engaging in counseling,” tailoring content for the intended audience, incorporating best practices to support comprehension, and enhancing clarity through simplified phrasing and the addition of concrete examples. Once approved, the materials underwent a structured translation process, guided by the World Health Organization’s (WHO’s) translation and adaptation guidelines [20]. These guidelines were selected because they are specifically designed for translating and adapting public health materials across diverse linguistic and cultural contexts. The WHO approach emphasizes conceptual equivalence rather than direct linguistic translation, ensuring that key health messages remain accurate, culturally appropriate, and accessible to target populations [20]. The translation process followed a 3-step approach, informed by key principles of the WHO translation and adaptation guidelines, with the goal of ensuring both cultural relevance and linguistic accuracy. First, bilingual members of our research team conducted the initial translation, prioritizing conceptual clarity over direct linguistic equivalence. Second, the translated materials underwent review by community-based organization advisers to evaluate cultural appropriateness and clarity. Third, the content was verified through an iterative process involving bilingual staff and outreach team members, who ensured alignment with the lived experiences and language norms of the target communities. To ensure linguistic and cultural appropriateness, we also cross-checked key terminology and phrasing with native Koreans living in South Korea, which helped confirm both accuracy and resonance with Korean cultural norms. This iterative process ensured that all dissemination materials were both accessible and contextually meaningful to our participants.

We anticipated that content that was both culturally respectful and carefully targeted would foster acceptance and engagement with new knowledge, motivating behavioral change. In alignment with cognitive behavioral theory [21], the project facilitated discussions around cultural perspectives and traditional practices related to dementia and caregiving. The KAB model is based on the cognitive behavioral theory, and the educational program helps participants reflect on their values, habits, needs, and wants and empowers them to explore and adopt desirable attitudes and behaviors about health care practices to improve their health. Following the dissemination activity, we aimed to motivate and empower participants to apply their newfound knowledge to their lives and caregiving after attending the training and educational workshops with experts and trusted leaders, or from reading the how-to-materials fact sheets.

#### Dissemination Activities

KIMCHI conducted in-person workshop presentations and online dissemination activities.

#### Workshop Presentations

Key findings from the studies by Smith [17] and Jennings et al [18] were synthesized and culturally adapted to resonate with the specific needs, values, and concerns of the Korean American community. Specifically, we incorporated Korean-language metaphors and idiomatic expressions to enhance relevance and comprehension and reflected culturally specific caregiving practices commonly observed in Korean American families, such as the value of filial piety and multigenerational caregiving structures. We also integrated religious and spiritual elements, including the influence of Christianity and ancestral rites, which shape caregiving beliefs and behaviors.

Moreover, insights from Somang Society’s dementia caregiver support groups informed our cultural adaptation process. Through their work with Korean American caregivers, they identified strategies used by older adults to conceal early signs of dementia due to stigma or fear of judgment. For example, individuals experiencing unusual forgetfulness may avoid admitting memory lapses by using general honorifics such as “Elder [Name]” instead of recalling specific names of fellow church members. This behavior reflects a broader cultural tendency to hide symptoms and reinforces the need for sensitive, community-informed approaches in addressing early detection and support.

All cultural adaptations were developed through iterative consultations with bilingual community partners and cultural experts to ensure the materials were accessible and meaningfully aligned with the lived experiences of Korean American participants. The workshop was facilitated by bilingual community partners from AARIN and the Somang Society, each holding a PhD—one in biomedical and biochemical engineering, the other in social work. Both facilitators possess extensive expertise in dementia and caregiving research and have demonstrated longstanding leadership in serving older Korean American communities. They were involved in the translation process to ensure that the content delivered was appropriate and understandable for an audience composed primarily of older Korean Americans, reviewed the key PCORI findings, collaborated with other partners in the network, and created culturally sensitive presentation slides to present to the participants.

To evaluate changes in KAB related to ADRD and cognitive aging, participants were asked to complete a pretest questionnaire before and after the workshop. Participant satisfaction was also evaluated through a brief survey. From March 2023 to May 2023, we conducted 11 workshops across 5 community sites: Korean Community Services of Metropolitan New York, Korean Community Services New Jersey, Chicago Hanul Family Alliance, Asian Women’s Christian Association New Jersey, and La Palma Church in California. Each workshop lasted approximately 1 hour. Participants spent 10-15 minutes completing pre- and postparticipation tests and an additional 5 minutes on a satisfaction survey.

#### Online Dissemination

### Fact Sheets

Two KIMCHI fact sheets, titled “Healthy Cognitive Aging” and “Caregiving for Loved Ones with Dementia,” were created in both English [22] and Korean [23] (Multimedia Appendices 1 [17,18] and 2 [17,18], respectively). These fact sheets aimed to empower the Korean American community with practical tips and comprehensive information on enhancing cognitive health and improving caregiving practices. The fact sheets were disseminated through various channels, including Somang Society’s printed newsletters, emails to participants in the Collaborative Approach for AANHPI Research and Education registry [14,15], and in-person at the Irvine Korean Cultural Festival. The 2-page flyer included a QR code and a link to the survey.

### Social Media Outreach

KIMCHI also established and used social media channels, including Facebook [24], X (formerly known as Twitter) [25], and YouTube [26], for dissemination. Messages were crafted to engage the Korean American community effectively, using culturally relevant content in both English and Korean to promote ongoing involvement with the KIMCHI project. This broad range of engagement styles aimed to increase the reach toward Korean Americans nationwide.

#### Ethical Considerations

We would like to clarify that the University of California, San Francisco institutional review board determined that this project is not a formal research study, but rather a program evaluation; thus, this project does not require institutional review board approval or informed consent. Nevertheless, data were securely collected and stored in University of California, San Francisco’s REDCap (Research Electronic Data Capture; Vanderbilt University), which is compliant with the Health Insurance Portability and Accountability Act. All 211 attendees of the KIMCHI workshop completed the pre- and posttest surveys and were awarded a gift card as appreciation for their participation and time. Online participants were also compensated with a gift card.

#### Measures

##### Sociodemographic Information

Participants’ self-reported demographic information was also collected, including age, sex, birthplace (including the year of arrival to the United States, if applicable), education, and household income. In addition, participants were asked to rate their proficiency in speaking, reading, and writing English on a 5-point Likert scale from “very well” to “not at all.” Participants who reported “some,” “a little bit,” or “not at all” were categorized as having limited English proficiency (LEP). Participants were also asked about their experience with individuals affected by ADRD, history of receiving education or training in dementia care, and interest in further learning about dementia.

##### KAB Survey

Pre- and posttest surveys on KAB regarding cognitive aging and caregiving were conducted to assess changes in participants’ KAB regarding cognitive aging and caregiving. The surveys consisted of 21 items based on validated questionnaires [27,28] as well as additional culturally appropriate items that were developed for the KIMCHI project (Textbox 2 [27,28]).

**Details of the Knowledge, Attitudes, and Behaviors pre- and postparticipation survey.**

**Knowledge**

- On the basis of the Alzheimer Disease Knowledge Scale from Carpenter et al [27], 9 knowledge component items were designed as true or false questions, and the instrument can be found in Multimedia Appendix 3 [27,28]. Knowledge item 1 was developed by the Koreans Invested in Making Caregivers’ Health Important (KIMCHI) team. The total sum score from the scale was added, where the higher score demonstrated greater knowledge related to Alzheimer disease and related dementias (ADRD). The standardized Cronbach α for the knowledge items in the pre- and postparticipation tests was 0.55 and 0.70, respectively.

**Attitude**

- Referencing the Dementia Attitudes scale-6 from Clark et al [28], 6 attitude items were rated on a 3-point Likert scale from “agree to neutral to disagree,” to assess participants’ feelings about caregiving for individuals with ADRD (Multimedia Appendix 3). The total sum score from the scale was computed, where the higher score yielded more positive attitudes regarding ADRD. The standardized Cronbach α for the attitude items in the pre- and postparticipation tests was 0.63 and 0.77, respectively.

**Behavior**

- Six behavior component items, rated on a 3-point Likert scale from “agree to neutral to disagree,” were used to assess participants’ engagement in activities related to cognitive aging and ADRD prevention (Multimedia Appendix 3). The behavior items were developed by the KIMCHI team and supported by the Patient-Centered Outcomes Research Institute advisers and the KIMCHI experts as appropriate to use. The total sum score from the scale was calculated, where the higher score showed greater positive engagement related to ADRD prevention and cognitive aging. The standardized Cronbach α for the behavior items in the pre- and postparticipation tests was 0.77 and 0.82, respectively.

After the workshop presentation, participants completed the same 21 items on KAB and a satisfaction survey (refer to the subsequent section). The standardized Cronbach α for the overall KAB items in the pre- and postparticipation tests was 0.70 and 0.79, respectively. Both surveys were not anonymized to connect participants’ pre- and posttest responses, and they were available in English and Korean, as well as electronic and paper formats. However, all participants opted to complete the survey in Korean and in paper format. Volunteers and staff members were available to assist participants with questions during the survey completion process.

##### Online Dissemination Satisfaction Survey

To evaluate participant experiences with the KIMCHI project’s educational materials, an online satisfaction survey, available in Korean and English, was administered and opened to anyone. We did not have a variable that collected participants’ language preference. This survey was designed to gather feedback on participants’ satisfaction with the dissemination activities, the relevance of the content, and their learning outcomes. The survey included questions assessing overall satisfaction, knowledge gained, and interest in future educational opportunities. Respondents rated their satisfaction with the workshop on a 5-point Likert scale from “very satisfied” to “very unsatisfied.” Participants were also asked if they learned something new from the program and if they were interested in participating in future community educational events related to cognitive aging, dementia caregiving, and advanced health care directives. For specific outreach channels where pretests were not feasible (eg, radio or social media), tailored questions were included. For Korean radio listeners, participants were asked about their understanding of ADRD prevention and care before and after listening to the program. Social media users were similarly inquired about their perceptions before and after engaging with content on platforms such as Facebook. Observations on what worked well and areas for improvement were discussed during the KIMCHI meetings that convened biweekly. The survey tool was administered to participants who engaged with the program through various platforms, including YouTube recordings, fact sheets, and social media posts. To check for the genuineness of entries, participants had to provide their email addresses and zip codes, and the KIMCHI team evaluated each survey to verify and ensure there were no duplicate entries from the same person. Each participant (via email) was provided with only one survey participation incentive. All online survey responses were collected, pooled, and analyzed to measure the effectiveness of the educational materials and content and identify areas for improvement.

#### Statistical Analysis

Data from the pretest and posttest questionnaires were analyzed to assess the effectiveness and significance of the KIMCHI educational community workshop presentations in improving participants’ KAB regarding ADRD prevention and dementia care. Because KAB has 3 possible categorical outcomes of either “true, false, or do not know” or “agree, neutral, or disagree,” we used Bhapkar test [29], which can be thought of as a generalization of McNemar test (which is only appropriate for dichotomous outcomes), to test whether there is marginal homogeneity between one or more of the row marginal proportions of the pretest and the column proportions of the posttest. Missing knowledge responses were included as “don’t know” in the analysis, and missing attitude and behavior responses were included as “neutral.” The pre- and posttest cores for knowledge were calculated as 2 points if answered correctly, 1 for do not know, and 0 for incorrect responses. Knowledge items 1, 3, 5, 6, and 9 were reverse-coded. In contrast, for attitude and behavior scores, 2 points were assigned for agree, 1 for neutral, and 0 for disagree responses. Total scores were combined in their perspective component to calculate the mean pre- and posttest scores. Overall weighted scores consider that the knowledge component has 3 more items than the attitudes and behaviors component, whereas all 3 subscales were equal in overall unweighted scores. Weighting was applied to ensure the total weighted score was contributed proportionally from each KAB item and prevent the knowledge items from being overpowered because it has 3 more items than the attitude and behavior items. Using paired samples *t* tests, we examined the overall mean changes in participants’ KAB and 95% CI regarding ADRD pre- and posttest scores. We used a Bonferroni corrected *P* value threshold to correct for multiple comparisons. As suggested by Cohen [30], we interpreted the effect size between the 2 sample means as small (*d*=0.2), medium (*d*=0.5), or large (*d*=0.8).

Overall, descriptive statistics summarized participants’ demographic characteristics. In contrast, inferential statistics, such as paired *t* tests, were used to compare pretest and posttest scores and assess the significance of any observed changes. To test for differences in the 5 pre-post score test components while controlling for additional factors, as a sensitivity analysis, we fit a linear mixed model for each of the test components with a random effect for individual and fixed effects for age, sex, LEP, education, and income. Subgroups with the highest number were the reference group. Statistical analyses were performed using SAS (version 9.4; SAS Institute Inc) software.

## Results

### In-Person Workshop Participant Characteristics

Table 1 presents the demographic characteristics of the KIMCHI participants. The average age of participants was 69 (SD 12.1) years, with 70.1% (148/211) of the participants identifying as female. The vast majority (203/211, 96.2%) were born outside of the United States and had LEP (167/211, 79.1%). More than half of the participants (117/211, 55.4%) had earned a bachelor degree or higher and had an annual household income of US $75,000 or less in the last year (104/211, 49.3%). In addition, more than a third of the participants (78/211, 37%) knew or worked with someone who has ADRD, and among them, 61% (47/77) had informal or family caregiving experience, and 39% (29/74) had professional experience. Notably, 83.4% (176/211) of the participants reported they had never received education or training in dementia care before.

**Table 1:** Overview of the sample characteristics among Koreans Invested in Making Caregivers' Health Important (KIMCHI) workshop participants (N=211).

Characteristics			Values
Age (y), mean (SD; range)			69 (12.1; 24-90)
**Age range (y), n (%)**			
	<50	12 (5.7)	
	50-59	30 (14.2)	
	60-69	52 (24.6)	
	70-79	70 (33.2)	
	≥80	40 (19)	
	Did not report	7 (3.3)	
**Sex, n (%)**			
	Female	148 (70.1)	
	Male	59 (28)	
	Did not report	4 (1.9)	
**Born in the United States, n (%)**			
	Yes	2 (0.9)	
	No	203 (96.2)	
	Did not report	6 (2.8)	
**If not born in the United States, year arrived in the United States (n=203), n (%)**			
	1925-1974	21 (10.3)	
	1975-1999	127 (62.6)	
	2000-2023	43 (21.2)	
	Did not report	12 (5.9)	
**Limited English proficiency^a^, n (%)**			
	Yes	167 (79.1)	
	No	35 (16.6)	
	Did not report	9 (4.3)	
**Education level, n (%)**			
	High school or less	44 (20.9)	
	Some college or technical school	40 (19)	
	Bachelor degree	80 (37.9)	
	Master degree or higher	37 (17.5)	
	Prefer not to answer	10 (4.7)	
**Annual household income (US $), n (%)**			
	≤25,000	41 (19.4)	
	25,001 to 75,000	63 (29.9)	
	75,001 to 150,000	40 (19)	
	≥150,001	21 (10)	
	Prefer not to answer	46 (21.8)	
“**Have you ever known or worked with someone who has Alzheimer disease or dementia,” n (%)**			
	Yes	78 (37)	
	No	130 (61.6)	
	Did not report	3 (1.4)	
“**I have informal or family caregiving experience**^b^**” (n=77), n (%)**			
	Yes	47 (61)	
	No	30 (39)	
“**I have professional caregiving experience**^b^**” (n=74), n (%)**			
	Yes	29 (39.2)	
	No	45 (60.8)	
“**Have you ever received education or training in dementia care,” n (%)**			
	Yes	33 (15.6)	
	No	176 (83.4)	
	Did not report	2 (0.9)	

^a”^No” refers to those who can speak, read, or write in English very well or well. “Yes” refers to those who can speak, read, or write some, a little bit, or not at all in English.

^b^Supplemental questions on informal or family caregiving and professional experiences were asked if participants answered “yes” to “Have you ever known or worked with someone who has Alzheimer’s disease or dementia?”

### Testing Marginal Homogeneity

Regarding the marginal homogeneity between the pre- and posttest responses, 48% (10/21) of the KAB items indicated significant differences (*P*<.002; Bonferroni correction for 21 tests; Table 2).

In the knowledge component, the posttest responses of 44% (4/9) of the survey items differed significantly from the pretest responses of the same participants: (1) “Having high blood pressure may increase a person’s risk of developing Alzheimer’s disease and related dementias” (*P*<.001), (2) “Once people have Alzheimer’s disease and related dementias, they are no longer capable of making informed decision about their own care” (*P*<.001), (3) “It has been scientifically proven that mental exercise can potentially help alleviate symptoms of Alzheimer’s disease and related dementias” (*P*<.001), and (4) “People whose Alzheimer’s disease and related dementias are not yet severe can benefit from psychotherapy for depression and anxiety” (*P*=.001).

In the attitudes component, the distribution of posttest responses in 50% (3/6) of the survey items differed significantly from the pretest responses of the same participants: (1) “People with Alzheimer’s disease and related dementias can be creative” (*P*<.001), (2) “It is possible to enjoy interacting with people with Alzheimer’s disease and related dementias” (*P*<.001), and (3) “People with Alzheimer’s disease and related dementias can enjoy life” (*P*<.001).

In the behaviors component, the distribution of posttest responses in 50% (3/6) of the survey items differed significantly from the pretest responses of the same participants: (1) “I engage in physical exercise to promote new brain cell growth” (*P*<.001), (2) “I engage in healthy diet to lower Alzheimer’s disease and related dementias risks” (*P*=.001), and (3) “I participate in research or other activities (dementia caregiving support group and/or classes) to learn more about Alzheimer’s disease and related dementias prevention and care” (*P*<.001).

The complete distribution of all 21 items with pre- and posttest KAB responses is displayed in Table 2.

**Table 2:** Distribution of Knowledge, Attitudes, and Behavior (KAB) survey responses, stratified by pre- and postparticipation tests, among workshop participants (N=211).

KAB questions				Pretest, n (%)		Posttest, n (%)		*P* value^a^	
**Knowledge**									
	**1. “Everyone develops dementia when he or she becomes old.”**								.50
		True	35 (16.6)		36 (17.1)				
		False	135 (64)		140 (66.4)				
		Do not know	41 (19.4)		35 (16.6)				
	**2. “Poor nutrition can make the symptoms of Alzheimer’s disease or dementia worse.”**								.003
		True	141 (66.8)		163 (77.3)				
		False	23 (10.9)		21 (10)				
		Do not know	47 (22.3)		27 (12.8)				
	**3. “When people with Alzheimer’s disease or dementia begin to have difficulty taking care of themselves, caregiver** **should take over right away.”**								.65
		True	171 (81)		170 (80.6)				
		False	17 (8.1)		21 (10)				
		Do not know	23 (10.9)		20 (9.5)				
	**4. “Having high blood pressure may increase a person’s risk of developing Alzheimer’s disease and related dementias.”**								*<.001* ^b^
		True	117 (55.5)		186 (88.2)				
		False	25 (11.9)		10 (4.7)				
		Do not know	69 (32.7)		15 (7.1)				
	**5. “Most people with Alzheimer’s disease and related dementias live in nursing homes.”**								.27
		True	69 (32.7)		60 (28.4)				
		False	99 (46.9)		107 (50.7)				
		Do not know	43 (20.4)		44 (20.9)				
	**6. “Once people have Alzheimer’s disease and related dementias, they are no longer capable of making informed decision about their own care.”**								*<.001*
		True	146 (69.2)		117 (55.5)				
		False	36 (17.1)		63 (29.9)				
		Do not know	29 (13.7)		31 (14.7)				
	**7. “It has been scientifically proven that mental exercise can potentially help alleviate symptoms of Alzheimer’s disease and related dementias.”**								*<.001*
		True	159 (75.4)		184 (87.2)				
		False	5 (2.4)		9 (4.3)				
		Do not know	47 (22.3)		18 (8.5)				
	**8. “People whose Alzheimer’s disease and related dementias are not yet severe can benefit from psychotherapy for depression and anxiety.”**								*.001*
		True	178 (84.4)		197 (93.4)				
		False	2 (0.9)		3 (1.4)				
		Do not know	31 (14.7)		11 (5.2)				
	**9. “Eventually, a person with Alzheimer’s disease and related dementias will need 24-hours supervision**.”								.04
		True	148 (70.1)		143 (67.8)				
		False	25 (11.9)		38 (18)				
		Do not know	38 (18)		30 (14.2)				
**Attitudes**									
	**10. “It is rewarding to care for people who have Alzheimer’s disease and related dementias.”**								.90
		Agree	163 (77.3)		166 (78.7)				
		Neutral	37 (17.5)		35 (16.6)				
		Disagree	11 (5.2)		10 (4.7)				
	**11. “I am comfortable touching people with Alzheimer’s disease and related dementias.”**								.30
		Agree	92 (43.6)		81 (38.4)				
		Neutral	91 (43.1)		100 (47.4)				
		Disagree	28 (13.3)		30 (14.2)				
	**12. “I feel relaxed around people with Alzheimer’s disease and related dementias.”**								.009
		Agree	15 (7.1)		24 (11.4)				
		Neutral	97 (46)		106 (50.2)				
		Disagree	99 (46.9)		81 (38.4)				
	**13. “People with Alzheimer’s disease and related dementias can be creative.”**								*<.001*
		Agree	33 (15.6)		55 (26.1)				
		Neutral	44 (20.9)		53 (25.1)				
		Disagree	134 (63.5)		103 (48.8)				
	**14. “It is possible to enjoy interacting with people with Alzheimer’s disease and related dementias.”**								*<.001*
		Agree	31 (14.7)		64 (30.3)				
		Neutral	90 (42.7)		83 (39.3)				
		Disagree	90 (42.7)		64 (30.3)				
	**15. “People with Alzheimer’s disease and related dementias can enjoy life.”**								*<.001*
		Agree	131 (62.1)		152 (72)				
		Neutral	46 (21.8)		43 (20.4)				
		Disagree	34 (16.1)		16 (7.6)				
**Behaviors**									
	**16. “I engage in cognitively stimulating everyday activities like reading, doing puzzles, learning a new skill or hobby.”**								.004
		Agree	119 (56.4)		139 (65.9)				
		Neutral	72 (34.1)		60 (28.4)				
		Disagree	20 (9.5)		12 (5.7)				
	**17. “I engage in physical exercise to promote new brain cell growth.”**								*<.001*
		Agree	109 (51.7)		137 (64.9)				
		Neutral	85 (40.3)		63 (29.9)				
		Disagree	17 (8.1)		11 (5.2)				
	**18. “I engage in healthy diet to lower Alzheimer’s disease and related dementias risks.”**								*.001*
		Agree	90 (42.7)		112 (53.1)				
		Neutral	97 (46)		82 (38.9)				
		Disagree	24 (11.4)		17 (8.1)				
	**19. “I engage in social activities to lower Alzheimer’s disease and related dementias risks.”**								.003
		Agree	85 (40.3)		105 (49.8)				
		Neutral	100 (47.4)		87 (41.2)				
		Disagree	26 (12.3)		19 (9)				
	**20. “I engage in positive thinking to maintain good mental health**.”								.95
		Agree	163 (77.3)		164 (77.7)				
		Neutral	45 (21.3)		43 (20.4)				
		Disagree	3 (1.4)		4 (1.9)				
	**21. “I participate in research or other activities (dementia caregiving support group and/or classes) to learn more about Alzheimer’s disease and related dementias prevention and care.”**								*<.001*
		Agree	51 (24.2)		72 (34.1)				
		Neutral	79 (37.4)		80 (37.9)				
		Disagree	81 (38.4)		59 (28)				

^a^*P* values were calculated with the Bhapkar chi-square test.

^b^Italicized *P* values indicate statistical significance (*P*<.002, a Bonferroni correction for 21 tests).

### Paired-Sample t Tests

As shown in Table 3, we observed a positive increase between the post- and prescores among the overall, weighted (t_210_=0.95, 95% CI 0.77-1.13) and unweighted (t_210_=0.91, 95% CI 0.74-1.09) KAB scores. Weighted and unweighted analyses led to the same conclusions. Specifically, in the knowledge summary score, the mean score was 1.17 (SD 2.29; 95% CI 0.86-1.48) points higher at the posttest than at the pretest. Participants collectively showed increased changes in their attitude summary score (t_210_=0.80, SD 2.22; 95% CI 0.50-1.10) at the posttest than at the pretest. Similarly, for the behavior summary score, the mean score was 0.76 (SD 2.03; 95% CI 0.49-1.04) points higher at the posttest than at the pretest. The knowledge component exhibited the highest average score in both the pre- and postparticipation tests, attributable to the inclusion of 9 knowledge items compared to 6 items each in the attitudes and behaviors components. There was a moderate effect size as the overall weighted and unweighted knowledge component between the pre- and posttest scores differed by 0.71 and 0.51 SDs, respectively. We detected small effect sizes in the components of attitudes (*d*=0.36) and behaviors (*d*=0.38). Through the paired *t* test analysis, all the mean changes and 95% CIs yielded a significance level of *P*<.001.

**Table 3:** Unweighted and weighted pre- and postsummary scores on overall knowledge, attitudes, and behaviors for workshop participants (N=211).

Survey	Items, n	Prescore, mean (SD; range)	Postscore, mean (SD; range)	*t* test (*df;* SD)	95% CI	Cohen *d*	*P* value^a^
Overall, weighted^b^	21	8.56 (1.75; 5-14)	9.51 (1.73; 6-14)	0.95, (210; 1.33)	(0.77-1.13)	0.71	*<.001*
Overall, unweighted^c^	21	8.26 (9.51; 4-13)	9.17 (1.73; 5-13)	0.91, (210; 1.29)	(0.74-1.09)	0.71	*<.001*
Knowledge	9	10.34 (2.67; 3-18)	11.51 (2.64; 6-18)	1.17, (210; 2.29)	(0.86-1.48)	0.51	*<.001*
Attitudes	6	6.33 (2.44; 0-12)	7.13 (2.83; 0-12)	0.80, (210; 2.22)	(0.50-1.10)	0.36	*<.001*
Behaviors	6	8.11 (2.68; 0-12)	8.88 (2.72; 1-12)	0.76, (210; 2.03)	(0.49-1.04)	0.38	*<.001*

^a^Italicized *P* values indicate statistical significance (*P*<.01; Bonferroni correction for 5 tests).

^b^Overall weighted scores consider that the knowledge component has 3 more items than attitudes and behaviors.

^c^Overall unweighted scores consider that all 3 subscales are equal.

In the linear mixed model results, the difference between the post- and pretest scores for the 5 test components remained unchanged, compared to the paired *t* test (Table 3), after adjusting for age, sex, LEP, education, and income (Multimedia Appendix 4).

### In-Person Workshop Satisfaction Survey

Table 4 presents the findings from the in-person participant workshop satisfaction survey. A notable 93.8% (196/209) of participants expressed satisfaction or high satisfaction with the workshop presentation, affirming that the topics, content, and presentation style were culturally relevant and applicable. Furthermore, 89.4% (185/207) of respondents indicated that they learned something new from this presentation and expressed interest in gaining further education on ADRD-related topics.

**Table 4:** In-person participant satisfaction survey following the dissemination activity (N=211).

Posttest survey questions		Participants, n (%)
“**Are you satisfied with the presenter’s presentation/workshop today” (n=209)**^a^		
	Very satisfied	98 (46.9)
	Satisfied	98 (46.9)
	Neutral	13 (6.2)
	Unsatisfied	0 (0)
	Very unsatisfied	0 (0)
“**The topics, content, and presentation style are culturally relevant and applicable” (n= 209)**^a^		
	Strongly agree	100 (47.8)
	Agree	96 (45.9)
	Neutral	12 (5.7)
	Disagree	1 (0.5)
	Strongly disagree	0 (0)
“**Did you learn something new from this presentation” (n=207)**^a^		
	Yes	185 (89.4)
	No	22 (10.6)
“**Are you interested in learning more about dementia” (n=209)**^a^		
	Yes	186 (89)
	No	23 (11)

^a^Totals may not add up to 211 due to missing values.

### Online Participant Characteristics and Satisfaction Survey

Table 5 displays the findings of the total nonpresentation or online evaluation survey (N=134) from the KIMCHI fact sheets (n=66, 49.3%), YouTube videos (n=63, 47%), and Facebook social media posts (n=5, 3.7%). The average age of the participants was 53 (SD 16.4) years; 64.7% (86/133) of the participants identified as female, while 35.3% (47/133) identified as male; and 79.9% (107/134) of the participants were born outside of the United States. Slightly more than half (75/134, 56%) of the participants demonstrated LEP in reading, writing, and speaking. Participants were also highly educated, with 82.7% (110/133) having a bachelor degree or higher and 46.3% (62/134) with a yearly household income of US $75,000 or higher. Regarding caregiving experiences, 46.6% (62/133) of the participants had known or cared for someone who had ADRD, and among this subgroup, 60% (37/62) had formal or family caregiving experiences, and 23% (14/62) had professional experiences. Although only 19.5% (26/133) of the participants had received education or training in dementia care, 79.1% (106/134) showed interest in learning more about dementia.

The overall satisfaction and content of the evaluation survey were encouraging. Of the 134 participants, 115 (85.8%) expressed that they were satisfied or very satisfied with the KIMCHI fact sheets, YouTube videos, and social media content on Facebook, and 87.2% (116/133) agreed or strongly agreed that the topics, content, and presentation style were culturally relevant and applicable. Finally, 82.1% (110/134) of the participants learned something new from the nonpresentation contents, and 77.6% (104/134) would be interested in learning more about future advanced directives on health, cognitive aging, and dementia caregiving.

Our social media outreach efforts capture significant reach, attention, and participation of our target audience. As of April 15, 2024, our Facebook page has garnered 426 viewers, while our Twitter (later rebranded as X) account has reached an audience of 2453 viewers. Our YouTube channel has attracted 1511 viewers and garnered at least 4390 total views. In addition, we have reached 100,000 listeners from the ethnic media, with these numbers representing total views. Finally, we have distributed 12,500 copies of fact sheets, exceeding our initial goal of 3000 copies, through community partner sites, newsletters, and in-person Korean cultural events.

**Table 5:** Sociodemographic characteristics of online participants (N=134).

Characteristics			Values
Age (y), mean (SD; range)			53 (16.4; 22-87)
**Age range (y), n (%)**			
	<40	33 (24.6)	
	40-49	28 (20.9)	
	50-59	22 (16.4)	
	60-69	22 (16.4)	
	≥70	29 (21.6)	
**Sex (n=133)** ^a^ **, n (%)**			
	Male	47 (35.3)	
	Female	86 (64.7)	
**Born in the United States, n (%)**			
	Yes	27 (20.1)	
	No	107 (79.9)	
**Year arrived in the United States (n=107), n (%)**			
	1919-1999	48 (44.9)	
	2000-2023	38 (35.5)	
	Did not report	21 (19.6)	
**Limited English proficiency, n (%)**			
	Yes	75 (56)	
	No	59 (44)	
**Education level (n=133)^a^, n (%)**			
	High school or less	12 (9)	
	Some college or technical school	11 (8.3)	
	Bachelor degree	70 (52.6)	
	Master degree or higher	40 (30.1)	
**Annual household income in the last year (US $), n (%)**			
	≤25,000	13 (9.7)	
	25,001 to 75,000	39 (29.1)	
	75,001 to 150,000	34 (25.4)	
	150,001 to 200,000	11 (8.2)	
	≥200,001	17 (12.7)	
	Prefer not to answer	20 (14.9)	
“**Have you ever known or cared for someone who has Alzheimer’s disease or dementia**^a^**” (n=133), n (%)**			
	Yes	62 (46.6)	
	No	71 (53.4)	
“**I have informal/family caregiving experience**^b^**” (n=62), n (%)**			
	Yes	37 (59.7)	
	No	25 (40.3)	
“**I have professional caregiving experience**^b^**” (n=62), n (%)**			
	Yes	14 (22.6)	
	No	48 (77.4)	
“**Have you ever received education or training in dementia care**^a^**” (n=133), n (%)**			
	Yes	26 (19.5)	
	No	107 (80.5)	
“**Do you have an interest in learning more about dementia,” n (%)**			
	Yes	106 (79.1)	
	No	28 (20.9)	
“**Are you satisfied with the KIMCHI**^c^ **fact sheets, YouTube video you watched or social media content you read,” n (%)**			
	Very satisfied	66 (49.3)	
	Satisfied	49 (36.6)	
	Neutral	16 (11.9)	
	Unsatisfied	3 (2.2)	
	Very unsatisfied	0 (0)	
“**The topics, content, and presentation style are culturally relevant and applicable**^a^**” (n=133), n (%)**			
	Strongly agree	52 (39.1)	
	Agree	64 (48.1)	
	Neutral	15 (11.3)	
	Disagree	2 (1.5)	
	Strongly disagree	0 (0)	
“**Did you learn something new from our fact sheets, YouTube video, or social media content,” n (%)**			
	Yes	110 (82.1)	
	No	24 (17.9)	
“**Do you want to learn more about future community educational or learning opportunities related to healthy cognitive aging, dementia caregiving, and advance healthcare directive, such as this research,” n (%)**			
	Yes	104 (77.6)	
	No	30 (22.4)	

^a^Totals do not add up to 134 due to missing values.

^b^Supplemental questions on informal or family caregiving and professional experiences were asked if participants answered “yes” to “Have you ever known or worked with someone who has Alzheimer’s disease or dementia?”

^c^KIMCHI: Koreans Invested in Making Caregivers’ Health Important.

## Discussion

### Principal Findings

The KIMCHI project disseminated the PCORI evidence on healthy cognitive aging and dementia care within the Korean American community through a variety of platforms, reaching a wide audience and achieving high participant engagement. The final dissemination effort included 11 in-person workshops, and attrition was a nonfactor as 211 participants who initially enrolled completed pre- and postsurveys. The results revealed significant improvements and changes in participants’ KAB (*P*<.001) regarding cognitive aging and caregiving. In addition, 134 online satisfaction surveys indicated exceptionally high satisfaction, with most respondents reporting newly acquired knowledge and a strong interest in future educational opportunities on cognitive aging and dementia caregiving.

The project’s multimodal dissemination approach—combining in-person presentations, ethnic media, and social media—effectively broadened its reach. Each method contributed to engagement as shown by comparative participation rates across platforms. The high satisfaction ratings, with 93.8% (196/209) and 85.8% (115/134) of respondents scoring 3 or higher on a 5-point Likert scale for in-person workshop and online dissemination, respectively, along with statistically significant changes in KAB scores (*P*<.001), underscore the project’s impact. Although we did not survey online participants with KAB items, social media and online resources provided valuable avenues for individuals who could not attend in-person presentations, ensuring the dissemination of critical information on cognitive health and caregiving practices.

In this project, cultural sensitivity refers to the deliberate effort to align content with the linguistic, social, and belief systems commonly observed among Korean American communities, particularly first-generation immigrants. To make ADRD content culturally appealing and meaningful, several adaptations were implemented. These included using Korean-language idioms and culturally familiar caregiving scenarios, integrating values, such as filial piety and respect for elders, and addressing stigma-related barriers, such as the perception of dementia as a natural part of aging or a source of family shame. We also incorporated religious and spiritual elements, such as Christian-based perspectives and ancestral rites, which are prevalent within segments of the Korean American population. Insights from bilingual community partners and organizations, such as the Somang Society and the AARIN, were instrumental in shaping the content. Their direct experiences with Korean American caregivers highlighted subtle yet impactful behaviors—such as avoiding the use of specific names to hide memory loss—that informed how we addressed stigma and early detection in a culturally responsive way.

The KIMCHI project highlights the impact of culturally adapted, community-based educational programs in addressing knowledge gaps and shifting attitudes and behaviors related to dementia caregiving among Korean Americans. The project’s findings resonate with previous research on health education in ethnic minority populations, suggesting that culturally and linguistically tailored programs are instrumental in reducing stigma and enhancing engagement in dementia care and prevention.

### Comparison With Previous Work

A limited number of previous studies have examined dementia-related KAB among Korean Americans. The KIMCHI project builds upon this foundation using a structured pre- and posttest KAB model to quantitatively assess changes within this population. KIMCHI differs from previous projects due to its larger sample size (or greater reach), bilingual resources, broader dissemination strategies, and same-day implementation of the pre- and postparticipation tests. This approach is consistent with research on culturally tailored programs, which have shown promise in enhancing dementia literacy among ethnic minority groups. For example, a pilot study [12] tested a dementia literacy intervention for Korean Americans with dementia and their caregivers, in which they assessed long-term knowledge retention, whereas KIMCHI measured same-day posttest changes, reporting a modest increase in dementia literacy scores by an average of 1 point (SD 2.4) at a 12-week follow-up. Similarly, the KIMCHI project observed a significant mean knowledge component change of 1.17 (SD 2.29; *P*<.001) in the pre- and postparticipation tests. These findings collectively underscore the importance of culturally responsive programs, such as KIMCHI, in enhancing dementia-related awareness and caregiving tools among Korean Americans.

Another relevant study [4] explored concerns and planning behaviors related to AD among Asian American subgroups and identified higher levels of concern and planning intent among Korean Americans compared to Chinese Americans. The study found Korean Americans had higher odds of worrying about developing AD (OR 2.65, 95% CI 1.86-3.79), having the possibility of caregiving for someone with AD (OR 1.60, 95% CI 1.16-2.23), and planning for future AD needs (OR 1.56, 95% CI 1.01-2.42) [4]. While previous research [4] found higher levels of AD concern, they did not assess actual engagement in their educational programs compared to the KIMCHI project, which used evaluation surveys after the workshop and digital platform engagement.

In addition, studies involving broader AANHPI communities have reported similar challenges and educational needs. One study [31] implemented a KAB focus group about healthy aging, ADRD, and dementia among older Chinese Americans (N=18) in New York City but did not conduct the pre- and postparticipation tests. The qualitative results disclosed their cultural beliefs about ADRD and dementia, how aging can lead to cognitive decline, and challenges to incorporating daily healthy diets, which were topics discussed in the KIMCHI presentations [31]. Another study [32] also focused on the knowledge, attitudes, and perceptions of ADRD and healthy aging among AANHPI communities without pre- and postparticipation tests. In their interview series with AANHPI community stakeholders (N=11), structural barriers to health care, cultural norms, and the lack of social support for AANHPI communities were identified, with a determination to provide culturally focused programs to combat these challenges, early detection, and ADRD prevention [32].

### Digital Technology Engagement

The KIMCHI project used digital platforms—social media, the project website, and ethnic media (radio and television)—to reach individuals who could not attend in-person workshops. The rationale for this broad range of engagement styles was to provide stakeholders with flexible access to ADRD resources; we accumulated over 4390 total social media views and 100,000 ethnic media viewers. We were unable to track the total number of fact sheet downloads on the participant’s end. This approach was particularly effective for individuals seeking to engage with materials at their convenience, including those who may have otherwise missed in-person presentations. To ensure continued accessibility, we plan to promote our KIMCHI resources, such as fact sheets [22,23] and presentation recordings, through our social media channels, including Facebook [24] and X (formerly known as Twitter) [25] pages, as well as YouTube videos [26]. Up-to-date content, event announcements, and future engagement opportunities will remain accessible on the KIMCHI website [33], ensuring a sustained digital presence (Table 5) and ongoing community engagement.

As a potential next step to explore in future work, we may consider designing a culturally and linguistically appropriate ADRD generative artificial intelligence tool for Korean American family caregivers. This potential next step can be guided by research with other heterogeneous older adult populations. For example, in a recent study [34], researchers tested a culturally relevant artificial intelligence mobile app for Black American informal caregivers and reported some positive findings, including an engaging and inclusive experience.

### Limitations

There are several limitations that should be considered when interpreting the findings. One limitation was the timing of the data collection, with pre- and postparticipation tests administered immediately before and after each workshop. This short interval may have limited participants’ ability to fully absorb and reflect on the information presented, potentially leading to an underestimation of immediate long-term understanding or behavior change. In addition, the timing of some workshops, particularly those scheduled after Sunday worship services, may have affected participation and attention levels, especially among older adults who may have been fatigued from an extended period of engagement. Future studies could adjust the timing of workshops to avoid such issues.

The KIMCHI project used a pre- and posttest survey approach to assess short-term changes in KAB among participants following dissemination activities. While this approach offered valuable quantitative insights, we acknowledge the limitations of not including qualitative methods (eg, focus groups or interviews) or a long-term follow-up component. However, these elements were not incorporated due to the defined scope and objectives of the project, which focused on outreach and education rather than formal research. As such, the design prioritized feasible, scalable data collection methods aligned with the project’s goals of raising awareness and gathering immediate community feedback.

We recognize the limitations of treating Korean Americans as a monolithic group. The Korean American community is diverse in terms of generation, immigration history, acculturation level, language proficiency, education, and religious beliefs. While our materials were primarily developed for older, first-generation Korean immigrants, we acknowledge that this approach may not fully capture the needs or perspectives of younger generations or more acculturated individuals. Future efforts should strive for a more segmented approach that reflects this intragroup diversity. Overall, while our culturally tailored approach was grounded in community input and relevant cultural frameworks, we recognize the importance of continued refinement and differentiation to reflect the full spectrum of experiences within the Korean American community.

Another limitation was the potential ambiguity of some KAB survey items and response bias. For example, knowledge item 3, “When people with Alzheimer’s disease or dementia begin to have difficulty taking care of themselves, the caregiver should take over right away*,*” may not have been clear in specifying whether it referred to professional support or family member caregivers. Because the survey was conducted in person with volunteers and staff members present, this may have led to social desirability bias in responses. The KIMCHI team reassured the participants that their responses would be kept confidential and protected. Future studies should refine survey items to ensure clarity and avoid misinterpretation and should not require participants to provide their names.

There are sociodemographic factors, such as marital status or health care access, that influence ADRD burden and the development of dementia. In the Health and Retirement Study [35], divorced (OR=2.05; *P*<.001), widowed (OR=1.52; *P*<.001), cohabiting (OR=1.55; *P*<.05), and never married (OR=1.6; *P*<.005) respondents had significantly higher odds of developing dementia than their married counterparts, after controlling for age, sex, race, education, and proxy report. Moreover, having access to health insurance could decrease the burden of ADRD and reduce mortality [36]. Limited studies have explored the link between marital status and health care access and ADRD knowledge, and future studies should investigate the associations.

We acknowledge that there may be a potential for ceiling effects, as some participants answered the knowledge component questions correctly. Among the 211 participants, 25 (11.8%) answered at least 78% (7/9) of the knowledge questions correctly in the pretest, while 3 (1.4%) participants had perfect scores. Although 2 of the 3 participants received perfect scores, they stated “Yes” to the “Did you learn something new from this presentation?” question in the posttest survey. Overall, most of the participants in the workshop did not have high baseline KAB scores, which did not limit the ability to detect improvements in knowledge or changes in attitudes and behaviors in the posttest.

In addition, inconsistencies in measurement scales may have influenced response patterns. While knowledge items were listed in the “true-false-do not know” format, attitudes and behaviors items were measured using a 3-point Likert scale: “agree-neutral-disagree.” This discrepancy may have affected comparability and response consistency across domains. Standardizing all KAB items to a uniform scale would enhance internal consistency and improve comparability in future studies.

The KIMCHI project could not provide comparative data on knowledge retention between in-person and online participants. This was one of the challenges in digital outreach as we did not implement the same pre- and posttest program for online participants to examine their KAB components after reading the fact sheets, watching our YouTube videos, or viewing our Facebook posts. The online participants did not have the opportunity to engage with our health care professionals and experts and ask any follow-up questions. The online participants were younger (mean age 53, SD 16.4 y) than the in-person workshop participants (mean age 69 years, SD 12.1), had less LEP (56% online vs 79% in-person), and had obtained a bachelor degree or higher (83% online vs 56% in-person). Studies that intend to conduct online surveys might foresee younger participants rather than older adults, particularly those with higher English proficiency, higher education (at least a bachelor’s degree), and more advanced technology skills.

Although fact sheets and social media generated an extensive reach in our online dissemination, our response rate regarding survey completion was low. With potential nonresponse bias, online participants were unable or unwilling to participate because they came across the fact sheets or social media posts randomly on their page and were unaware of what KIMCHI is about. In addition, web page surveys could lead to spam risks, as we received surveys (n=23) that were not genuine due to duplicates of the same responses in multiple surveys or contact information of non-Korean names. Therefore, we could not filter out the total view count to those that were genuine, which could be one reason the total views were higher than the response rate. Another reason for the low response rate is that online respondents need basic computer skills or an internet connection to complete the survey. Nonetheless, future studies should ensure they accurately differentiate between genuine and spam survey respondents when analyzing results. In this project, we presented the descriptive statistics of the online dissemination data as a comparison group to our workshop participants instead of making inferences. Finally, community-based and convenience sampling limits the generalizability of our findings and may not be representative of broader populations with different demographic or socioeconomic characteristics. Future studies should consider strategies to enhance the representativeness of the sample.

Despite these limitations, there are several notable strengths. One of our project’s strengths is to evaluate trends and patterns in KAB in the tests before and after participation in the workshops. In addition, the culturally tailored dissemination activities facilitated social engagement and fostered greater community participation, making the program more accessible and impactful. Importantly, the evaluation findings will contribute to refining future KIMCHI dissemination strategies, improving implementation processes, and enhancing the effectiveness of community educational dissemination activities on cognitive aging and dementia caregiving.

### Conclusions

The KIMCHI project’s community-based, culturally adapted dissemination model demonstrated effectiveness in changing KAB regarding healthy cognitive aging and caregiving among Korean Americans, in addition to increasing the willingness to learn more about ADRD and being involved in PCORI type of projects. By leveraging PCORI-funded research through a culturally tailored approach, caregivers and stakeholders were empowered to adopt new caregiving strategies that align with traditional Korean values of family and community support. Health care systems are encouraged to consider adopting similar culturally sensitive strategies to promote health equity and access to dementia-related resources for underserved populations. Future studies should continue to explore the efficacy of tailored programs, incorporating real-life case studies and other culturally relevant elements to enhance outcomes and strengthen the representation of Korean Americans in health research.

## Appendix

Multimedia Appendix 1 Online fact sheet for English readers.

Multimedia Appendix 2 Online fact sheet for Korean readers.

Multimedia Appendix 3 Survey items on knowledge, attitudes, and behaviors.

Multimedia Appendix 4 Results of the linear mixed effects analysis for the weighted and unweighted pre- and posttest summary scores on knowledge, attitudes, and behaviors (N=211).

## Abbreviations

AANHPI: Asian Americans, Native Hawaiians, and Pacific Islanders
AARIN: Asian American Resource and Information Network
AD: Alzheimer disease
ADRD: Alzheimer disease and related dementias
KAB: knowledge, attitudes, and behaviors
KIMCHI: Koreans Invested in Making Caregivers’ Health Important
LEP: limited English proficiency
PCORI: Patient-Centered Outcomes Research Institute
REDCap: Research Electronic Data Capture
WHO: World Health Organization
