# Supplementary material for: Knowledge, Attitudes, and Behaviors Related to Dementia Prevention and Caregiving Among Korean Americans (the KIMCHI Project): Pre- and Posttest Evaluation Study
Source: JMIR Aging. 2025 Aug 15;8:e72147. doi: 10.2196/72147 (PMC12397760; doi:10.2196/72147)
Supplement: Multimedia Appendix 1 [file aging_v8i1e72147_app1.pdf]

Caregiving for Loved Ones with Dementia

Kimchi
Koreans Invested in Making Caregivers Health Important

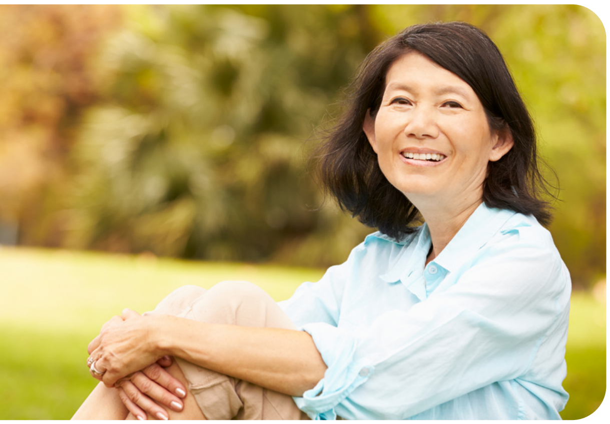

Koreans **I**nvested in **M**aking **C**aregivers **H**ealth **I**mportant (**KIMCHI**) is an education and outreach project on different health topics tailored to the Korean American culture for caregivers and other stakeholders that serve Korean Americans.

This fact sheet summarizes the dementia caregiving research to share tips for being a caregiver for a loved one with Alzheimer's or dementia.

Five Domains of Dementia Care

**Medical care**	**Physical quality of life**	**Social and emotional quality of life**	**Access to services and supports**	**Caregiver support**
Such as specialty care and pain management	Such as mobility and fall risk	Such as participation in pleasurable activities	Such as advance care planning	Such as caregiver support groups

Main Concerns

For persons with early-stage dementia

- Importance of engaging in meaningful activities
- Understand that the caregiver may need to adapt according to the level of functioning of the care recipient
- There are concerns that the care recipients are being a burden to their loved ones

For dementia caregivers

- Ensuring safety of care recipients
- Managing caregiver stress

Goals related to caregiving need to be redefined and readdressed as the disease progresses and it is common for caregivers and care recipients to have different opinions.

Korean American caregivers need more community support and dementia education opportunities tailored to their culture

Scan this QR code or click the link below to participate in the evaluation survey. A small incentive will be provided upon completion.

http://tiny.ucsf.edu/KIMCHISurveyFS

For more information about KIMCHI and outreach and educational resources, please contact the study team with any questions or concerns you have about this study at 415-498-0580 or email to kimchi@ucsf.edu.

https://kimchi.ucsf.edu/

Reference: Jennings, L. A., Palimaru, A., Corona, M. G., Cagigas, X. E., Ramirez, K. D., Zhao, T., Hays, R. D., Wenger, N. S., & Reuben, D. B. (2017). Patient and caregiver goals for dementia care. Quality of life research : an international journal of quality of life aspects of treatment, care and rehabilitation, 26(3), 685–693. https://doi.org/10.1007/s11136-016-1471-7

KIMCHI was funded through a Patient-Centered Outcomes Research Institute (PCORI) Eugene Washington PCORI Engagement Award EACB 24814.

AARIN Asian American Resource and Information Network
소망 소사이어티 SOMANG SOCIETY
UCSF University of California San Francisco

Healthy Cognitive Aging

Kimchi
Koreans Invested in Making Caregivers Health Important

https://kimchi.ucsf.edu/

Koreans **I**nvested in **M**aking **C**aregivers **H**ealth **I**mportant (**KIMCHI**) is an education and outreach project on different health topics tailored to the Korean American culture for caregivers and other stakeholders that serve Korean Americans.

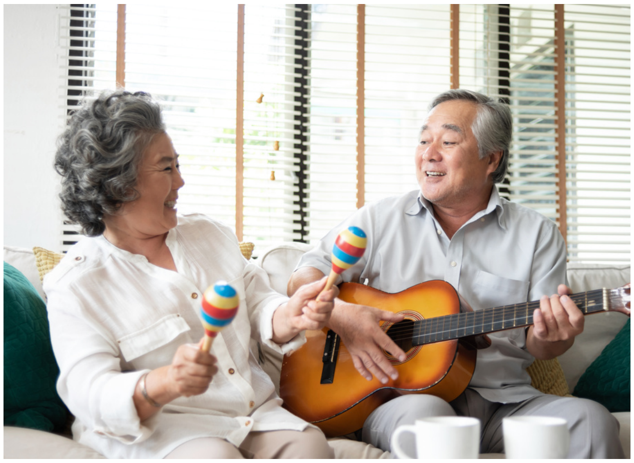

This fact sheet summaries key points from research about healthy cognitive aging so that you and your loved ones can learn more about brain health and prevent Alzheimer's disease and dementia.

As we age, we all experience some loss of our cognitive functioning. However, some people experience more significant cognitive declines beyond age-related changes and more consistent with a dementia process.

Alzheimer's disease and related dementias Risk Factors

- Unhealthy diet
- Lack of physical activity
- Lack of cognitive engagement
- Substance use
- Social isolations
- Sleep difficulties
- Mental health problems
- High blood pressure
- High cholesterol
- Diabetes
- Obesity
- Inflammatory disease

Promote healthy cognitive aging through

- Moderate-intensity exercises (brisk walking, dancing, and biking)
- Healthy and balanced diet
- Cognitive stimulation, training, and rehabilitation programs
- Pleasant activities and social engagement
- Cognitive behavioral therapy to better regulate our negative emotions, thoughts, and behaviors

Keep our brain stimulated just like how we keep our body active!

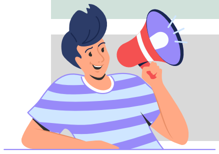

In general, anything **good for the heart** is **good for the brain!**

Take a holistic approach to improve your overall quality of life including your physical, psychological, social aspects!

For more information about KIMCHI and outreach and educational resources, please contact the study team with any questions or concerns you have about this study at 415-498-0580 or email to kimchi@ucsf.edu.

Scan this QR code or click the link below to participate in the evaluation survey. A small incentive will be provided upon completion.

http://tiny.ucsf.edu/KIMCHISurveyFS

Reference: Smith G. E. (2016). Healthy cognitive aging and dementia prevention. The American psychologist,71(4), 268–275. https://doi.org/10.1037/a0040250

KIMCHI was funded through a Patient-Centered Outcomes Research Institute (PCORI) Eugene Washington PCORI Engagement Award EACB 24814.

AARIN
소망 소사이어티 SOMANG SOCIETY
UCSF University of California San Francisco
