# Supplementary material for: Knowledge, Attitudes, and Behaviors Related to Dementia Prevention and Caregiving Among Korean Americans (the KIMCHI Project): Pre- and Posttest Evaluation Study
Source: JMIR Aging. 2025 Aug 15;8:e72147. doi: 10.2196/72147 (PMC12397760; doi:10.2196/72147)
Supplement: Multimedia Appendix 2 [file aging_v8i1e72147_app2.pdf]

치매 돌봄 관리

Kimchi
Koreans Invested in Making Caregivers Health Important

김치 (**K**oreans **I**nvested in **M**aking **C**aregivers **H**ealth **I**mportant-**KIMCHI**)는 알츠하이머나 치매 환자들을 보살피는 한인 간병인들과 가족 및 여러 이해 관계자를 위해 다양한 건강 주제를 한인문화에 맞춰 제공하는 교육•홍보 프로젝트입니다.

본 자료는 알츠하이머 및 치매 돌봄 관리 요령을 공유하기 위해 최신 치매 간병 연구자료를 한국어로 요약한 것입니다.

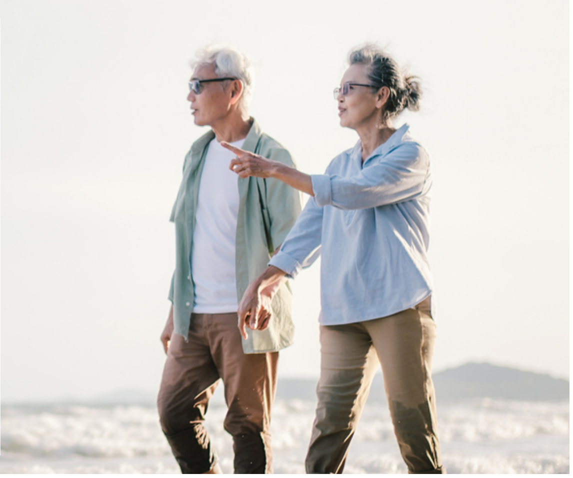

치매 관리의 5가지 영역

- **의료적 관리** 예. 전문 진료나 통증 관리
- **신체적 삶의 질** 예. 기동성 및 낙상 위험
- **사회적 감정적 삶의 질** 예. 즐거운 활동/모임 참여
- **서비스 및 지원에 대한 접근** 예. 사전 의료 계획
- **간병인 지원** 예. 간병인 서포트 그룹 모임

주요 우려사항들

- **치매 초기 환자들의 경우**
  - 의미 있는 활동 참여의 중요성
  - 간병 대상자가 스스로를 사랑하는 사람들에게 짐/부담이 된다고 생각하는 것에 대한 우려
  - 간병인들이 간병 대상자 개개인의 인지 기능 수준에 적응해야 할 필요성
- **치매 간병인의 경우**
  - 간병 대상자의 안전 확보
  - 간병에 따른 스트레스 관리

간병과 관련된 목표는 질병이 진행됨에 따라 재고되어야 하며, 때로는 간병인과 간병 대상자들 사이에 의견 차이가 있을 수 있습니다.
한인간병인에게 지역사회 지원과 한인 문화를 고려한 치매 교육이 절실히 필요합니다.

김치프로젝트의 홍보 및 교육 활동에 대해 더 자세한 정보를 원하시거나, 질문이나 우려사항이 있으시면 저희 연구팀 전화 415-498-0580 또는 이메일 kimchi@ucsf.edu로 연락을 주십시요.

QR 코드를 스캔하시거나, 아래 링크를
눌러 평가 설문 조사에 참여해주세요.
완료 시 소정의 인센티브가 제공됩니다.
http://tiny.ucsf.edu/KIMCHISurveyFS

레퍼런스: Jennings, L. A., Palimaru, A., Corona, M. G., Cagigas, X. E., Ramirez, K. D., Zhao, T., Hays, R. D., Wenger, N. S., & Reuben, D. B. (2017). Patient and caregiver goals for dementia care. Quality of life research : an international journal of quality of life aspects of treatment, care and rehabilitation, 26(3), 685–693. https://doi.org/10.1007/s11136-016-1471-7

KIMCHI 프로젝트는 Patient-Centered Outcomes Research Institute (PCORI) Eugene Washington PCORI Engagement Award EACB 24814가 후원합니다.

https://kimchi.ucsf.edu

AARIN 소망 소사이어티 SOMANG SOCIETY UCSF University of California San Francisco

Kimchi
Koreans Invested in Making Caregivers Health Important

뇌건강

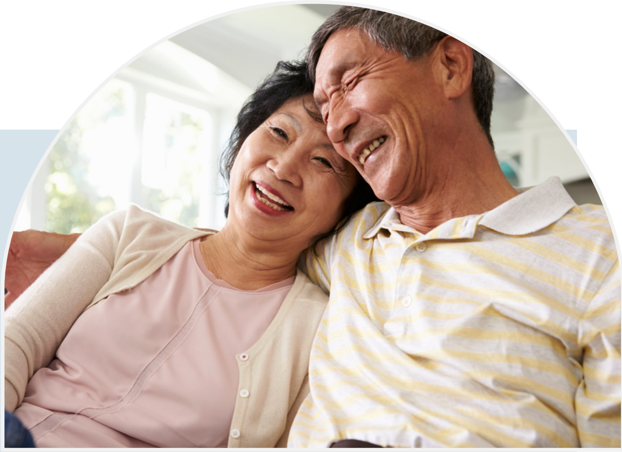

김치 (**K**oreans **I**nvested in **M**aking **C**aregivers **H**ealth **I**mportant-**KIMCHI**)는 알츠하이머나 치매 환자들을 보살피는 한인 간병인들과 가족 및 여러 이해 관계자를 위해 다양한 건강 주제를 한인문화에 맞춰 제공하는 교육•홍보 프로젝트입니다.

본 자료는 건강한 인지노화에 대한 최신 연구를 요약하여 본인 및 사랑하는 가족들이 뇌건강과 알츠하이머 및 치매예방에 대해 잘 알 수 있도록 돕기 위함입니다.

나이가 들면서 우리 모두는 어느 정도의 인지 기능의 상실을 경험합니다. 하지만, 어떤 사람들은 연령에 따른 변화보다 더 심각한 인지 저하를 경험하며 이는 치매로 진행되는 과정입니다.

알츠하이머병 및 치매와 관련된 위험 요소들

- 비건강식
- 신체활동 부족
- 뇌활동 부족
- 약물 사용
- 사회적 고립
- 수면 부족
- 정신건강 문제
- 고혈압
- 높은 콜레스테롤
- 당뇨병
- 비만
- 염증성 질환

뇌건강을 증진시키는 습관

- 하루 30분 이상 중간 강도의 신체활동
- 건강하고 균형 잡힌 식단
- 기억력 운동이나 문제해결 게임과 같은 뇌 자극, 훈련 및 재활
- 즐거운 활동 및 사회 참여
- 부정적인 감정, 생각, 행동을 바로 잡기 위한 상담 참여

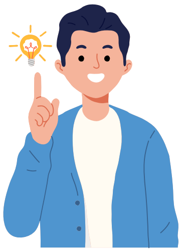

일반적으로 심장에 좋은 것은 뇌에도 좋습니다!
우리의 몸을 활동적으로 움직이는 것처럼 우리의 뇌도 자극해주세요!

신체적, 정신적, 사회적 측면을 포함한 전반적인 삶의 질을 향상시키기 위하여 뇌건강에 대한 총체적인 접근이 중요합니다!

https://kimchi.ucsf.edu

김치프로젝트의 홍보 및 교육 활동에 대해 더 자세한 정보를 원하시거나, 질문이나 우려사항이 있으시면 저희 연구팀 전화 415-498-0580 또는 이메일 kimchi@ucsf.edu로 연락을 주십시요.

QR 코드를 스캔하시거나, 아래 링크를 눌러 평가 설문 조사에 참여해주세요. 완료 시 소정의 인센티브가 제공됩니다.

http://tiny.ucsf.edu/KIMCHISurveyFS

레퍼런스: *Smith G. E. (2016). Healthy cognitive aging and dementia prevention. The American psychologist, 71(4), 268–275. https://doi.org/10.1037/a0040250*

KIMCHI 프로젝트는 Patient-Centered Outcomes Research Institute (PCORI) Eugene Washington PCORI Engagement Award EACB 24814가 후원합니다.

AARIN Asian American Resource and Information Network · 소망 소사이어티 SOMANG SOCIETY · UCSF University of California San Francisco
