# Supplementary material for: Knowledge, Attitudes, and Behaviors Related to Dementia Prevention and Caregiving Among Korean Americans (the KIMCHI Project): Pre- and Posttest Evaluation Study
Source: JMIR Aging. 2025 Aug 15;8:e72147. doi: 10.2196/72147 (PMC12397760; doi:10.2196/72147)
Supplement: Multimedia Appendix 3 [file aging_v8i1e72147_app3.docx]

Please answer these questions to the best of your ability BEFORE/AFTER participating in today's presentation.

Below are some statements about dementia and dementia caregiving. Please read each statement carefully and indicate whether you think the statement is True or False, to the best of your knowledge.

|  | True | False | Don’t know |
| --- | --- | --- | --- |
| 1. Everyone develops dementia when he or she becomes old. |  |  |  |
| 2. Poor nutrition can make the symptoms of Alzheimer's disease or dementia worse. |  |  |  |
| 3. When people with Alzheimer's disease or dementia begin to have difficulty taking care of themselves, caregiver should take over right away. |  |  |  |
| 4. Having high blood pressure may increase a person's risk of developing Alzheimer's disease and related dementias. |  |  |  |
| 5. Most people with Alzheimer's disease and related dementias live in nursing homes. |  |  |  |
| 6. Once people have Alzheimer's disease and related dementias, they are no longer capable of making informed decision about their own care. |  |  |  |
| 7. It has been scientifically proven that mental exercise can potentially help alleviate symptoms of Alzheimer's disease and related dementias. |  |  |  |
| 8. People whose Alzheimer's disease and related dementias are not yet severe can benefit from psychotherapy for depression and anxiety. |  |  |  |
| 9. Eventually, a person with Alzheimer's disease and related dementias will need 24-hours supervision. |  |  |  |

**Alzheimer’s Disease Knowledge Scale retrieved from Carpenter et al. (2009). doi: 10.1093/geront/gnp023.*

Please rate each statement according to how much you agree or disagree with it. There are no right or wrong answers.

|  | Agree | Neutral | Disagree |
| --- | --- | --- | --- |
| 1. It is rewarding to care for people who have Alzheimer's disease and related dementias. |  |  |  |
| 2. I am comfortable touching people with Alzheimer's disease and related dementias. |  |  |  |
| 3. I feel relaxed around people with Alzheimer's disease and related dementias. |  |  |  |
| 4. People with Alzheimer's disease and related dementias can be creative. |  |  |  |
| 5. It is possible to enjoy interacting with people with Alzheimer's disease and related dementias. |  |  |  |
| 6. People with Alzheimer's disease and related dementias can enjoy life. |  |  |  |

* *Dementia Attitudes Scale-6 from Clark et al. (2023). doi: 10.1177/00914150221106094.*

Please indicate to what extent you agree with following statements.

|  | Agree | Neutral | Disagree |
| --- | --- | --- | --- |
| 1. I engage in cognitively stimulating everyday activities like reading, doing puzzles, learning a new skill or hobby. |  |  |  |
| 2. I engage in physical exercise to promote new brain cell growth. |  |  |  |
| 3. I engage in healthy diet to lower Alzheimer's disease and related dementias risks. |  |  |  |
| 4. I engage in social activities to lower Alzheimer's disease and related dementias risks. |  |  |  |
| 5. I engage in positive thinking to maintain good mental health. |  |  |  |
| 6. I participate in research or other activities (dementia caregiving support group and/or classes) to learn more about Alzheimer's disease and related dementias prevention and care. |  |  |  |

References

27. Carpenter, BD, Balsis, S, Otilingam, PG, Hanson, PK, Gatz, M. The Alzheimer's Disease Knowledge Scale: development and psychometric properties. Gerontologist. 2009;49(2): 236–247. PMID:19363018. doi.org/10.1093/geront/gnp023

28. Clark MS, Ebert AR, Hicks Patrick J. The DAS-6: A Short Form of the Dementia Attitudes Scale. International Journal of Aging and Human Development. 2023;96(4):488-500. PMID:35656738. doi:10.1177/00914150221106094
