# Supplementary material for: Knowledge, Attitudes, and Behaviors Related to Dementia Prevention and Caregiving Among Korean Americans (the KIMCHI Project): Pre- and Posttest Evaluation Study
Source: JMIR Aging. 2025 Aug 15;8:e72147. doi: 10.2196/72147 (PMC12397760; doi:10.2196/72147)
Supplement: Multimedia Appendix 4 [file aging_v8i1e72147_app4.docx]

| **Supplemental table.** Sensitivity analysis results of linear mixed model of sociodemographic variables on weighted, unweighted, knowledge, attitudes, and behaviors pre- and postsummary scores (N=211). | | | | | | | | | | | |
| --- | --- | --- | --- | --- | --- | --- | --- | --- | --- | --- | --- |
| Variable | | Estimate (95% CI) | *P* value^a^ | Estimate (95% CI) | *P* value^a^ | Estimate (95% CI) | *P* value^a^ | Estimate (95% CI) | *P* value^a^ | Estimate (95% CI) | *P* value^a^ |
|  | | Overall, weighted^b^ | | Overall, unweighted^c^ | | Knowledge | | Attitudes | | Behaviors | |
| **Post- vs. Prescore** | | 0.95 (0.77, 1.13) | *<.001* | 0.92 (0.74, 1.09) | *<.001* | 1.17 (0.86, 1.48) | *<.001* | 0.80 (0.50, 1.10) | *<.001* | 0.76 (0.49, 1.04) | *<.001* |
| **Age range (years)** | | | | | | | | | | | |
|  | <50 years old | 1.27 (0.26, 2.28) | .014 | 1.15 (0.13, 2.18) | .028 | 2.02 (0.58, 3.45) | *.006* | 3.01 (1.52, 4.49) | *<.001* | -1.57 (-3.20, 0.07) | .060 |
|  | 50-59 years old | 0.96 (0.26, 1.66) | *.008* | 0.76 (0.04, 1.47) | .038 | 2.00 (1.00, 3.00) | *.000* | 1.19 (0.16, 2.23) | .024 | -1.20 (-2.33, -0.06) | .040 |
|  | 60-69 years old | 0.52 (-0.04, 1.08) | .069 | 0.42 (-0.15, 0.99) | .145 | 1.15 (0.36, 1.95) | *.005* | 0.28 (-0.54, 1.10) | .504 | -0.19 (-1.10, 0.71) | .672 |
|  | 70-79 years old | Reference | | Reference | | Reference | | Reference | | Reference | |
|  | ≥80 years old | -0.01 (-0.65, 0.63) | .965 | 0.07 (-0.58, 0.72) | .829 | -0.45 (-1.36, 0.46) | .332 | 0.46 (-0.48, 1.40) | .336 | 0.33 (-0.71, 1.37) | .530 |
|  | Did not report | 0.07 (-1.76, 1.90) | .941 | 0.13 (-1.73, 1.98) | .893 | -0.28 (-2.89, 2.32) | .830 | 0.34 (-2.35, 3.02) | .805 | 0.14 (-2.82, 3.10) | .926 |
| **Sex** | | | | | | | | | | | |
|  | Female | Reference | | Reference | | Reference | | Reference | | Reference | |
|  | Male | -0.03 (-0.52, 0.47) | .918 | -0.10 (-0.60, 0.40) | .698 | 0.41 (-0.30, 1.12) | .252 | -0.08 (-0.81, 0.65) | .826 | -0.64 (-1.45, 0.16) | .115 |
|  | Did not report | 0.78 (-2.17, 3.73) | .602 | 0.51 (-2.49, 3.50) | .740 | 2.27 (-1.94, 6.47) | .289 | 1.41 (-2.93, 5.74) | .523 | -2.43 (-7.21, 2.34) | .316 |
| **Limited English proficiency^d^** | | | | | | | | | | | |
|  | Yes | Reference | | Reference | | Reference | | Reference | | Reference | |
|  | No | 0.75 (0.15, 1.34) | .014 | 0.81 (0.21, 1.41) | *.009* | 0.36 (-0.48, 1.21) | .396 | 0.95 (0.08, 1.83) | .032 | 1.13 (0.17, 2.09) | .021 |
|  | Did not report | -0.02 (-1.46, 1.42) | .977 | -0.11 (-1.35, 1.57) | .884 | -0.91 (-2.96, 1.14) | .383 | 0.05 (-2.06, 2.16) | .963 | 1.18 (-1.14, 3.51) | .317 |
| **Education level** | | | | | | | | | | | |
|  | High school or less | -0.07 (-0.67, 0.53) | .819 | -0.005 (-0.61, 0.60) | .987 | -0.41 (-1.27, 0.44) | .340 | 0.18 (-0.70, 1.06) | .687 | 0.23 (-0.74, 1.20) | .646 |
|  | Some college or technical school | -0.29 (-0.90, 0.31) | .339 | -0.20 (-0.81, 0.42) | .527 | -0.77 (-1.63, 0.09) | .078 | 0.55 (-0.34, 1.44) | .224 | -0.32 (-1.30, 0.66) | .521 |
|  | Bachelor's degree | Reference | | Reference | | Reference | | Reference | | Reference | |
|  | Master's degree or higher | 0.36 (-0.27, 0.98) | .259 | 0.38 (-0.25, 1.01) | .241 | 0.33 (-0.56, 1.21) | .467 | 0.48 (-0.43, 1.40) | .297 | 0.30 (-0.70, 1.31) | .553 |
|  | Prefer not to answer | -0.39 (-1.74, 0.96) | .570 | -0.29 (-1.67, 1.08) | .673 | -0.63 (-2.56, 1.30) | .520 | 0.52 (-1.47, 2.50) | .609 | -0.73 (-2.91, 1.46) | .514 |
| **Annual household income (US$)** | | | | | | | | | | | |
|  | ≤25,000 | -0.22 (-0.85, 0.40) | .484 | -0.14 (-0.77, 0.50) | .670 | -0.75 (-1.64, 0.14) | .100 | 0.31 (-0.61, 1.23) | .507 | -0.11 (-1.12, 0.90) | .836 |
|  | 25,001 to 75,000 | Reference | | Reference | | Reference | | Reference | | Reference | |
|  | 75,001 to 150,000 | -0.19 (-0.83, 0.46) | .569 | -0.13 (-0.79, 0.52) | .687 | -0.59 (-1.50, 0.33) | .207 | 0.34 (-0.61, 1.28) | .481 | -0.33 (-1.37, 0.71) | .527 |
|  | ≥150,001 | 0.11 (-0.70, 0.92) | .791 | 0.11 (-0.71, 0.93) | .789 | 0.13 (-1.03, 1.28) | .829 | 0.28 (-0.91, 1.47) | .646 | 0.02 (-1.30, 1.33) | .981 |
|  | Prefer not to answer | -0.39 (-1.01, 0.23) | .218 | -0.40 (-1.03, 0.23) | .216 | -0.35 (-1.24, 0.53) | .434 | -0.18 (-1.09, 0.74) | .705 | -0.74 (-1.75, 0.26) | .146 |
| ^a^Italicized *P* values indicate statistical significance (*P*<.01, a Bonferroni correction for 5 tests).  ^b^Overall weighted scores consider that knowledge component has three more items than attitudes and behaviors.  ^c^Overall unweighted scores consider that all three subscales are equal.  ^d^”No” refers to those who can speak, read, or write in English very well or well. “Yes” refers to those who can speak, read, or write some, a little bit, or not at all in English. | | | | | | | | | | | |
